# Surgical Technique: Treatment of Distal Humerus Nonunions

**DOI:** 10.1007/s11420-017-9551-y

**Published:** 2017-04-12

**Authors:** Johanna C. E. Donders, Dean G. Lorich, David L. Helfet, Peter Kloen

**Affiliations:** 1 Department of Orthopedic Surgery, Academic Medical Centre, University of Amsterdam, Meibergdreef 9, Amsterdam, 1105 AZ The Netherlands; 2 Orthopaedic Trauma Service, Hospital for Special Surgery, 535 East 70th Street, New York, NY 10021 USA; 3 Weill Cornell Medical College, New York, NY 10065 USA

**Keywords:** nonunion, distal humerus, bone graft, internal fixation

## Abstract

**Background:**

Open reduction and internal fixation of distal humerus fractures is standard of care with good to excellent outcome for most patients. However, nonunions of the distal humerus still occur. These are severely disabling problems for the patient and a challenge for the treating physician. Fortunately, a combination of standard nonunion techniques with new plate designs and fixation methods allow even the most challenging distal humeral nonunion to be treated successfully.

**Questions/Purposes:**

The purpose of this manuscript is to describe our current technique in treating distal humeral nonunion as it has evolved over the last four decades. We have now follow-up on 62 treated patients.

**Methods:**

A few key steps are essential to obtain bone healing while regaining or preserving elbow motion. These include careful planning, extensile exposure, release of the ulnar nerve, capsular release and mobilization of the distal fragment, debridement, and finally stable fixation after alignment with application of bone graft.

**Results:**

The vast majority of distal humeral nonunions can be treated successfully with open reduction and internal fixation.

**Conclusion:**

Important components of the treatment plan are careful preoperative planning, extensile approach, debridement, and solid fixation with—locking—plates and liberal use of bone graft.

**Electronic supplementary material:**

The online version of this article (doi:10.1007/s11420-017-9551-y) contains supplementary material, which is available to authorized users.

## Introduction

Most often, a distal humerus nonunion is located at the supracondylar level with the articular fragments having healed in a near-anatomic position. Motion at the nonunion site causes pain, limited elbow function, and disability [1–4, 8–12, 21–23]. Hardware will ultimately fail or loosen with often a windshield wiper effect of the screws in the bone, further compromising bone stock [9, 10]. The increased motion at the supracondylar level, excessive scar formation, and inflammation around the ulnar nerve can lead to nerve symptoms including pain, numbness, and/or paresthesias. Use of the arm for loaded activities and positioning the forearm and hand against gravity will be severely compromised.

**Fig. 1:**
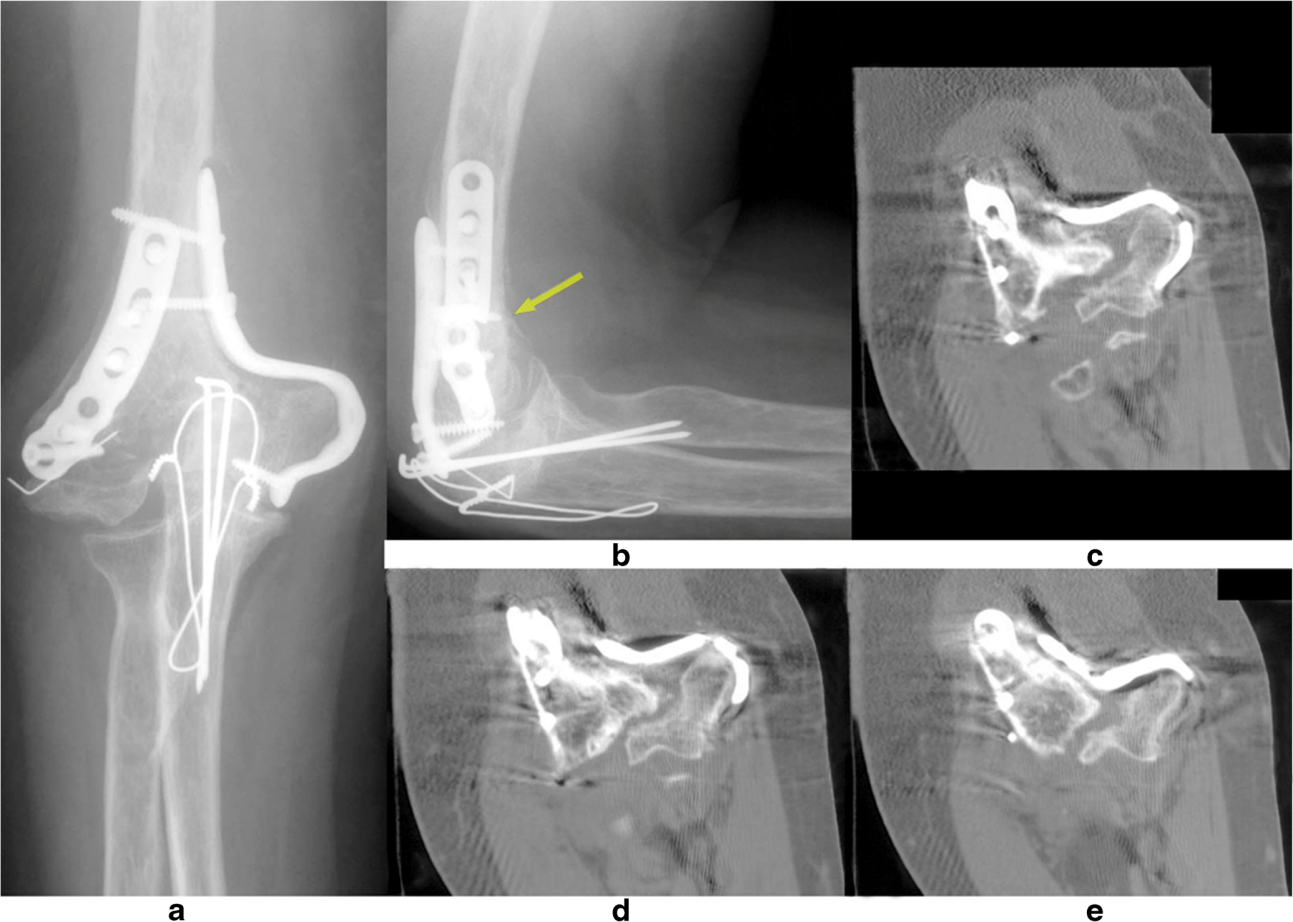
A 60-year-old female presents 8 months following ORIF of a right-sided distal humeral fracture with complaints of pain at the fracture site and limited range of motion of the elbow. Radiographs (**a**, **b**) and CT scan images (**c**–**e**) reveal a nonunion, and loss of fixation with plate breakage (medial side).

**Fig. 2:**
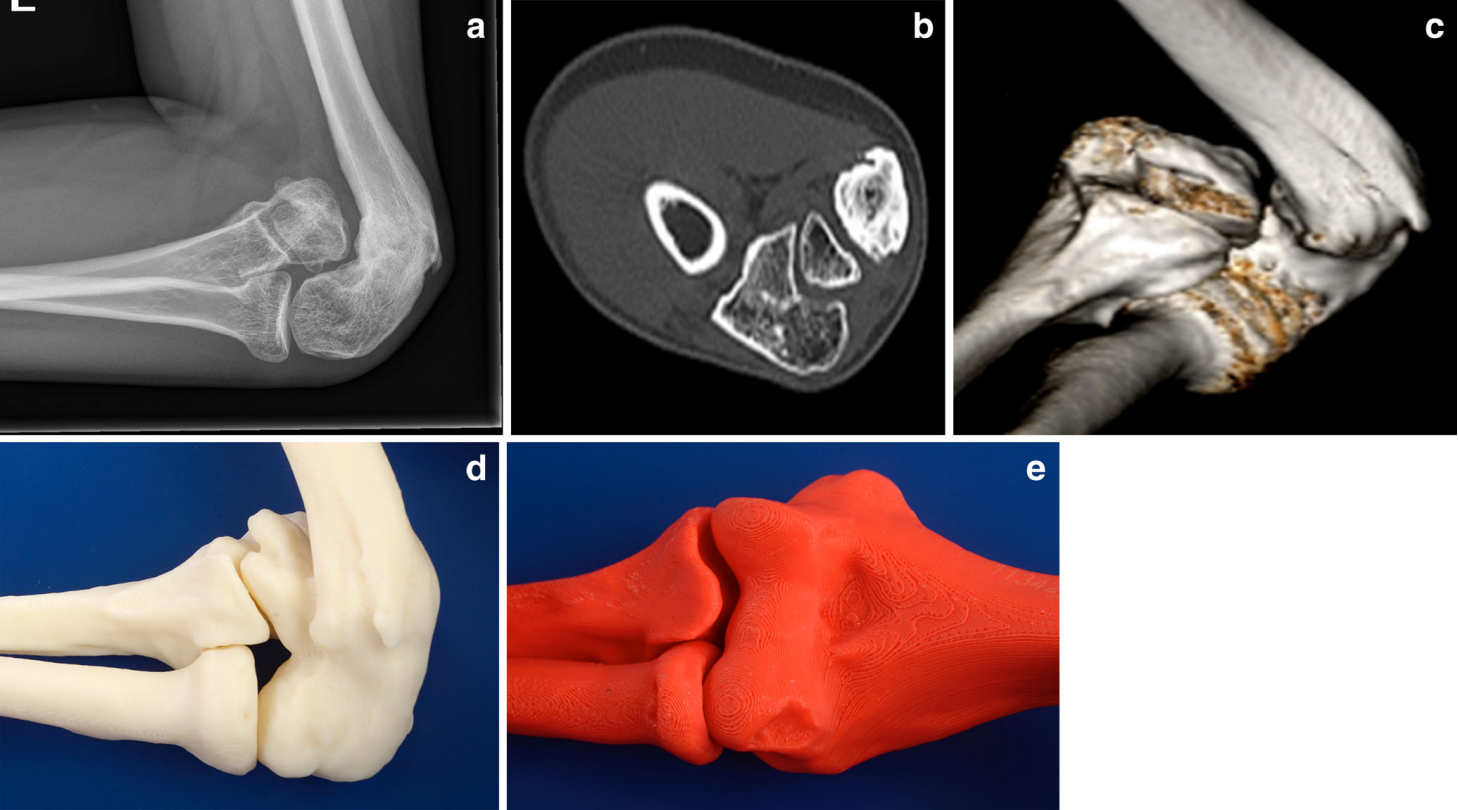
For complex cases, a 3D CT and 3D models of the affected and the mirror-imaged healthy elbow will provide better insight in the nonunion and associated deformity. This patient had a war-related injury to his elbow as a child and presented 11 years later. Plain radiographs suggested an elbow dislocation as seen on the lateral radiograph (**a**). 2D CT imaging (**b**) and 3D-reformatted CT imaging (**c**) showed a malunion of the distal humerus with associated medial condyle nonunion. In addition, there was overgrowth of the radial head and capitellum. The proximal radio-ulnar joint, the radio-capitellar joint, and the relation between the proximal ulna and medial condyle nonunion are intact. 3D-printed models of the affected (**d**) and mirror-imaged healthy side (*red*) (**e**) provide valuable insight.

A nonunion of the distal humerus is often oligotrophic, being a combination of decreased biological activity and insufficient stability. They can be sub-classified based on location being supracondylar, transcondylar, intercondylar, unicondylar (medial or lateral), or osteochondral [13, 19]. The size of the distal fragment can be underestimated as it is often flexed on the AP-radiograph. A CT scan will help determine the actual size (Fig. 1) [10]. In addition, for complex cases, we nowadays print 3D models of the nonunion and compare it to the mirror-imaged healthy side to help planning (Fig. 2). It is always important to consider an associated infection, even if there are no clear symptoms as fever, drainage, or wound problems.

### Treatment Options

Elbow arthrodesis, distraction arthroplasty [14], allograft [24], open reduction and internal fixation [1–4, 8–12, 16, 19, 20, 22, 23], prosthetic replacement [6, 7, 12, 15, 17, 18], and the Ilizarov technique [5, 20] have all been used in the management of distal humeral nonunions. Each of these treatment options has limitations and complications, including difficult bone fusion (due to restricted bone stock), inconsistent outcomes, instability, weakness and loss of function of the elbow joint, neuropathic joints, and infection.

Our experience is that most nonunions of the distal humerus can be treated surgically to obtain bone healing with a functional range of motion of the elbow*.* We described in 2002 what, to the best of our knowledge, is the largest series of 52 distal humeral nonunions treated with internal fixation [9]. Since this publication, new anatomic locking plates have been introduced allowing better fixation of small osteopenic nonunion fragments. The aim of this manuscript is to describe our current treatment of a distal humeral nonunion using a standardized treatment plan that includes careful planning, extensile exposure, release of the ulnar nerve, capsular release and mobilization of the distal fragment, debridement, and finally stable fixation after alignment with application of bone graft. We also provide a summary of the pertinent literature.

## Methods

### Surgical Technique

#### Preparation and Positioning

**Fig. 3:**
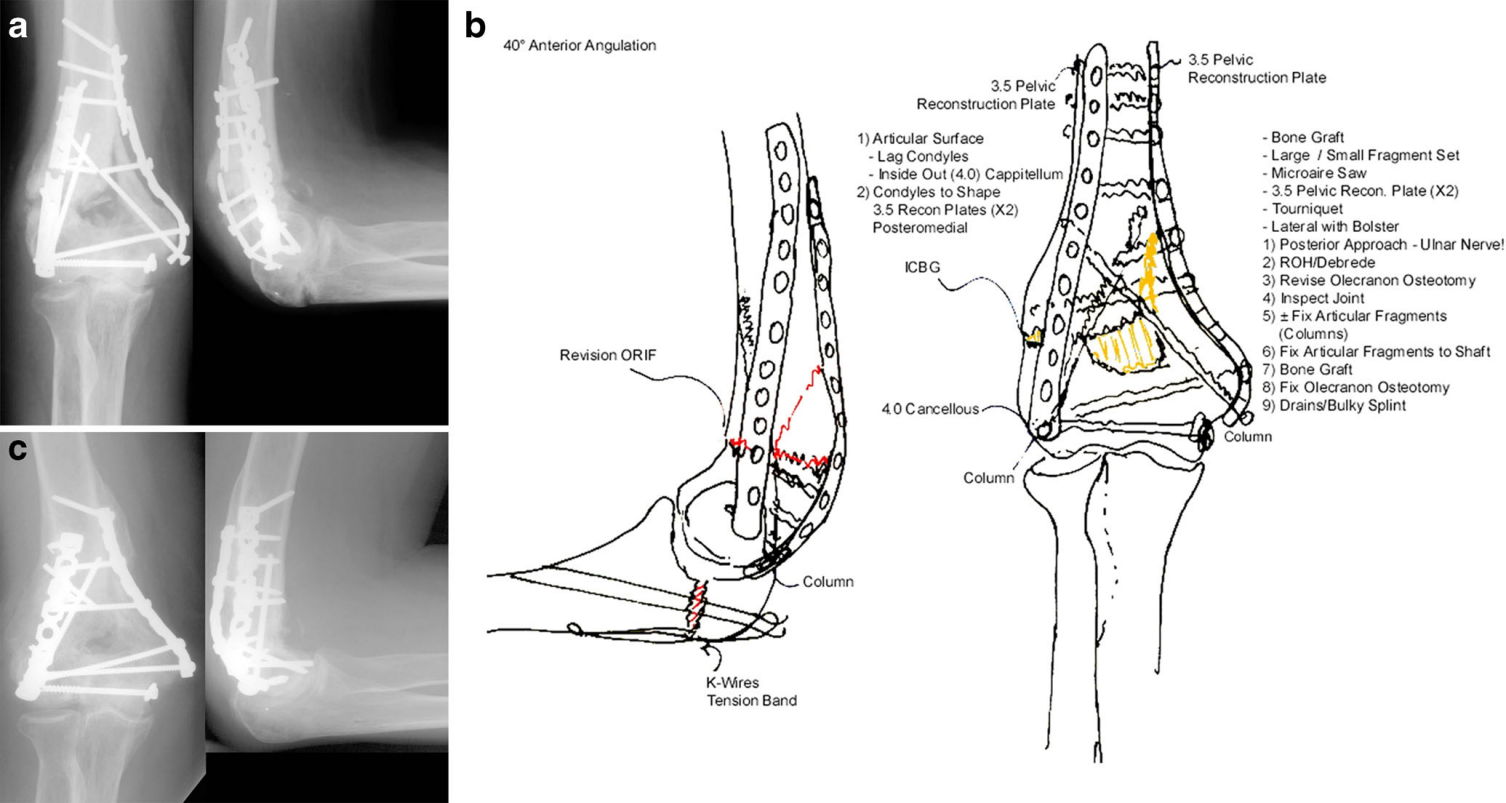
Good preoperative planning will greatly facilitate the surgical procedure. Preoperative anteroposterior (AP) and lateral radiographs (*from left to right*) show a distal humerus nonunion with failed hardware (**a**), detailed pre-op plan (**b**), and final AP and lateral radiographs (*from left to right*) illustrating a healed distal humerus nonunion (**c**).

The patient is positioned prone or in the lateral decubitus position. In the presence of an osteochondral shearing-type nonunion, a lateral approach might be better. A preoperative drawing outlining the surgical tactic will help anticipate and prevent intraoperative problems (Fig. 3). The patient is positioned supine with the arm abducted on an arm-table. No antibiotics are given until a minimum of five deep cultures are obtained.

#### Exposure

**Fig. 4:**
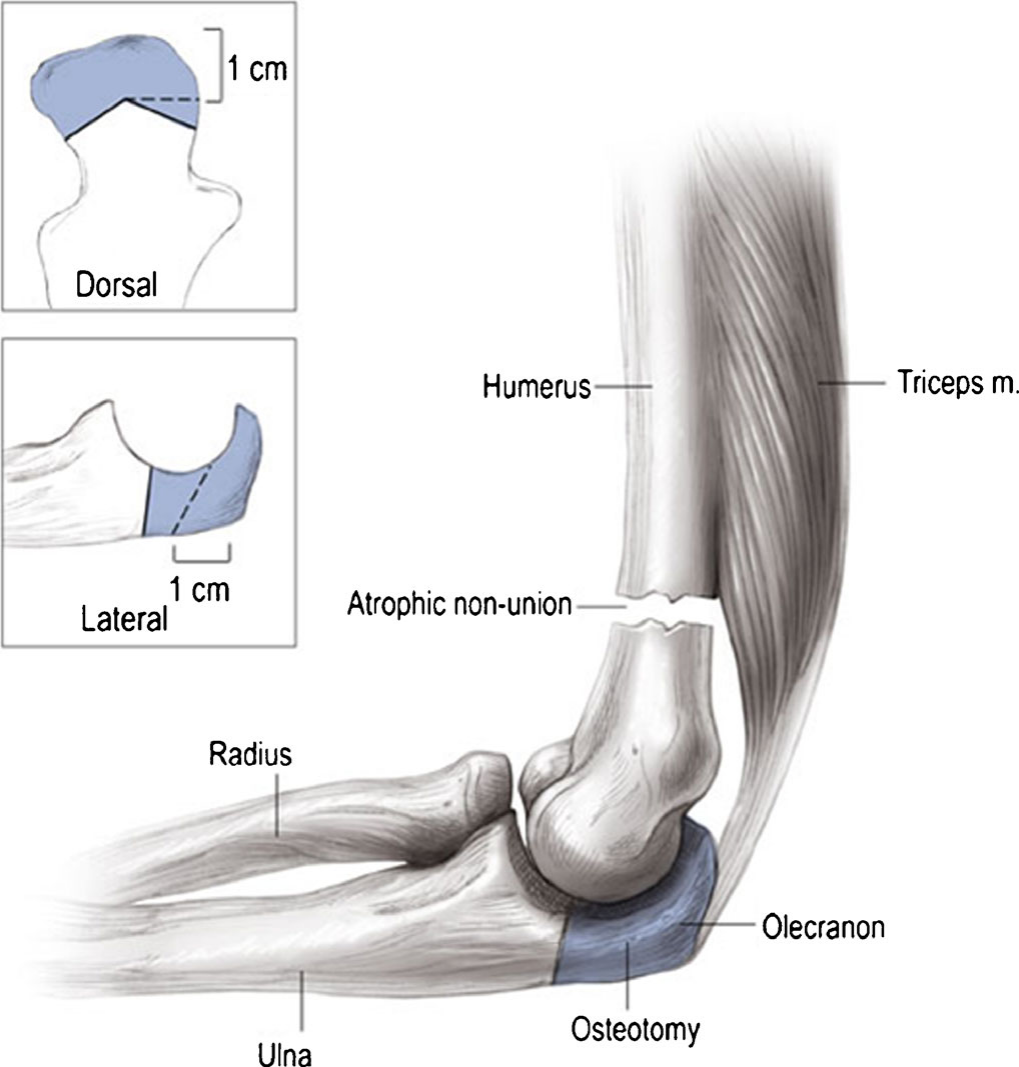
The olecranon osteotomy is angulated (as shown in *inset*), forming an apex to facilitate reduction and providing additional rotational stability for fixation (from: Helfet DL, Kloen P, Anand N, Rosen HS. ORIF of delayed unions and nonunions of distal humerus fractures. Surgical technique. J Bone Joint Surg Am. 2004;suppl 1:18–29. Reprinted with permission from The Journal of Bone and Joint Surgery, Inc).

**Fig. 5:**
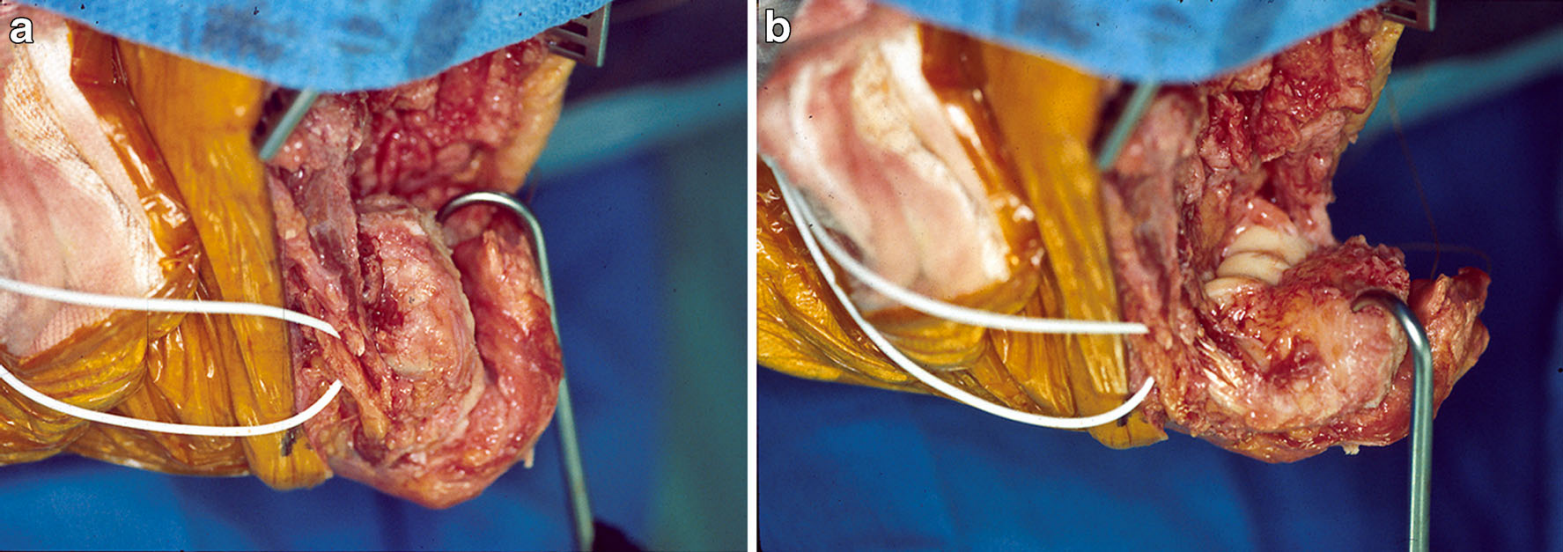
Intraoperative photographs showing the amount of motion of the distal fragment after an extensile release.

The posterior approach allows elevation of medial and lateral skin flaps and an olecranon osteotomy. The ulnar nerve is identified proximally and traced towards the elbow. If ulnar nerve symptoms are present preoperatively, external neurolysis is performed. The olecranon osteotomy for an intra-articular, transcondylar, or a low distal humeral nonunion provides superb exposure and ability for extensile release (Fig. 4). For a supracondylar distal humeral nonunion, a triceps splitting, paratricipital, or triceps-reflecting anconeus pedicle approach can be used. For a unicondylar nonunion, a medial or lateral approach is often sufficient. Alternatively, one can start with a paratricipital approach and add an olecranon osteotomy as needed. The chevron olecranon osteotomy is done at the semilunar notch where the cartilage is thinnest. Careful elevation of the proximal fragment prevents articular separation that can be the result of adherence of the olecranon cartilage to the trochlea. The triceps is elevated off the bone with a rasp of by finger dissection. All unstable hardware is removed. Nonunion tissue is debrided sharply. After clearing scar tissue and posterior capsule, the anterior capsule and scar can be released allowing increased joint mobilization (Fig. 5). If motion remains limited, the lateral collateral ligament can be osteotomized, allowing the joint to be hinged open on the medial ligamentous structures for further release.

#### Fixation

**Fig. 6:**
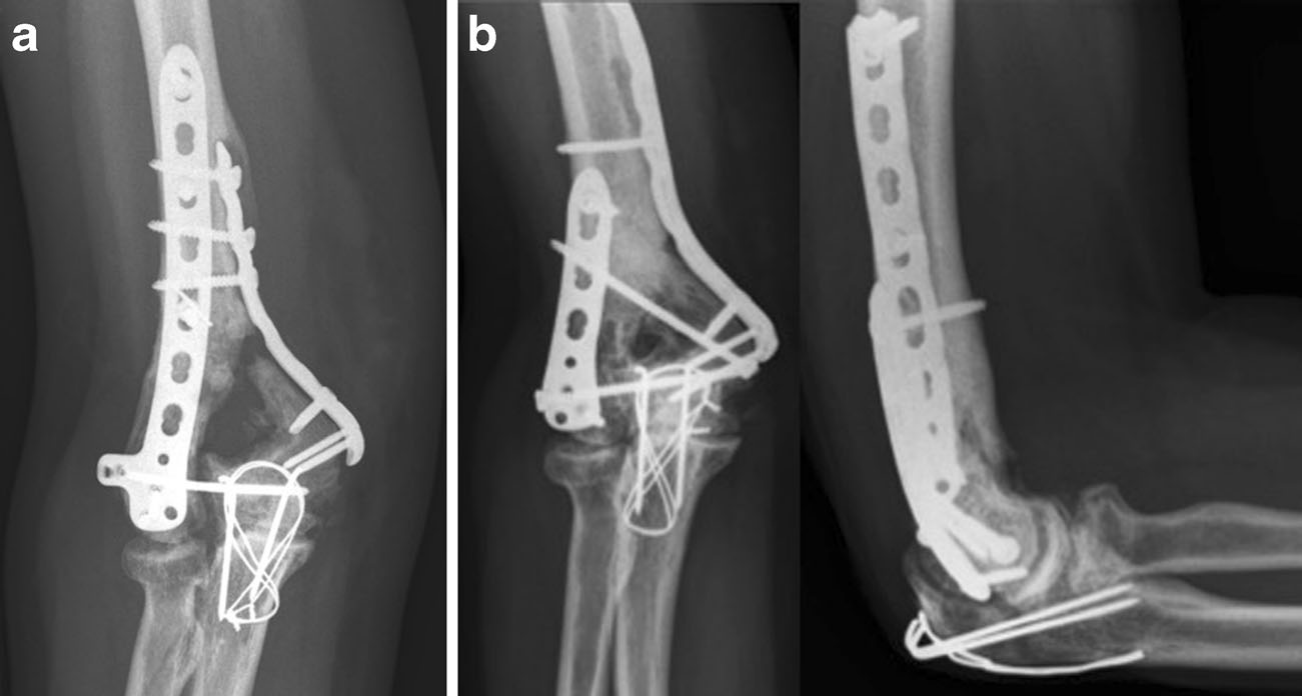
Anatomic locking plates (3.5 mm proximal and 2.7 mm distal) provide an increased number of fixation options. This patient had a nonunion of her distal humerus fracture that showed positive cultures for *Enterobacter cloacae* (**a**). Revision internal fixation with new plates bone graft and antibiotics resulted in healing as demonstrated on AP and lateral radiographs (*from left to right*); (**b**).

**Fig. 7:**
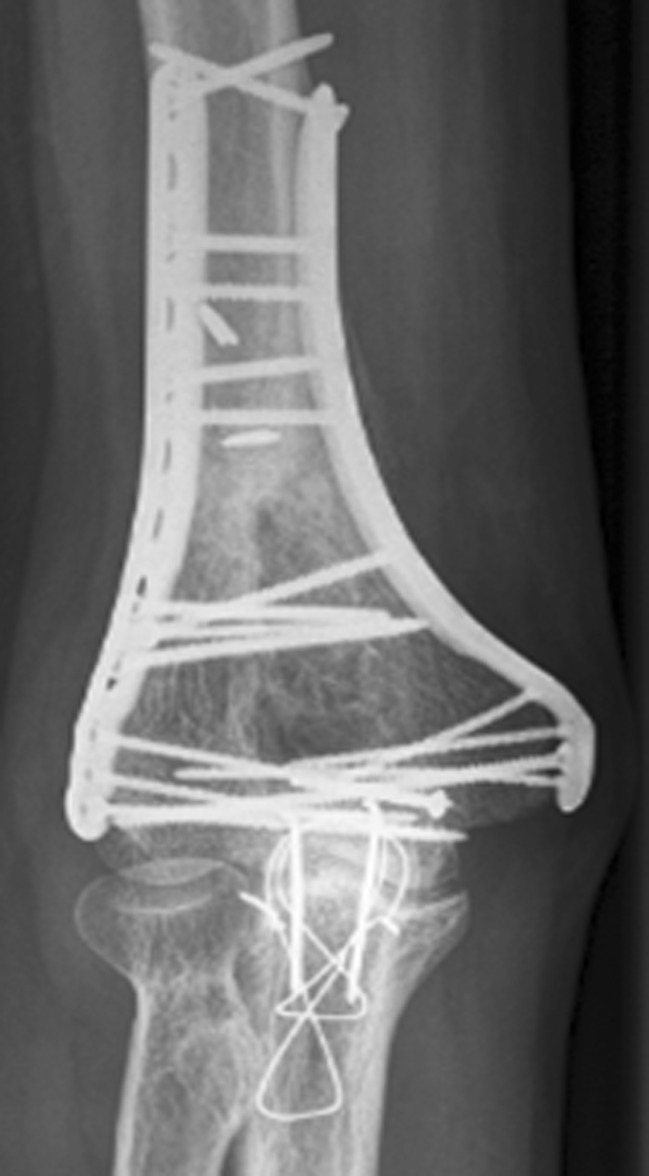
Using parallel plating, there can be “crowding” of the screws distally. Cross threading of these screws might actually increase the holding power of the fixation.

With a 2.0-mm drill bit, the medullary canal is opened on both sides of the nonunion until blood is seen to egress from the canal. Realignment and stable reduction when anatomy is distorted can be facilitated by creating a trough in the distal fragment into which the shaft is impacted. Position of the distal segment in relation to the shaft should be carefully checked to assure restoration of the “carrying angle” in the anteroposterior plane and the flexion condyles in the lateral plane. Occasionally, a malunion of the condyles is present that impedes motion. Corrective osteotomy can realign the intra-articular component. All fragments are temporarily reduced with K-wires. The condylar block is reduced to the shaft with two crossed K-wires. Definitive fixation is performed with plates. The changed anatomy will often preclude use of anatomic plates, thus requiring customized fixation. Locking plates nowadays allow better and more versatile fixation with 3.5-mm screws along the shaft and metaphysis and 2.7-mm peri-articular screws (Fig. 6). To maximize fixation stability, we prefer placing two long screws from distal thru the plates crossing from medial to lateral and from lateral to medial. Sometimes, there is screw crowding precluding easy drilling. With the oscillating drill mode, the drill often will find its way. Subsequent cross threading of screws (intentional or non-intentional) will actually increase the holding power of the screws (Fig. 7). Tension band fixation can augment fixation in osteopenic bone as it relies on muscle and ligament attachment to bone and not so much on bone quality. Autologous bone graft is the gold standard for nonunion treatment. With a chisel, small bone cuts are made on both sides of the nonunion, leaving soft tissues attached as much as possible and bone graft is added. The olecranon osteotomy is fixed with a figure-of-eight tension band or tension band plates with an intramedullary screw. If the ulnar nerve had preoperative symptoms, anterior nerve transposition can be performed. The elbow is placed in a removable splint. Gentle-active and active-assisted range of motion exercise is immediately started under guidance of a physical or occupational therapist, allowing light functional activities as pain allows. Healing is generally seen at 3–5 months after index surgery. Once the nonunion is healed, the patient is allowed unrestricted activities.

## Results

We evaluated 62 patients with a delayed or nonunion of the distal humerus. All patients were treated using the protocol as outlined above. Over the years, fixation devices have been modified and improved. The concepts of debridement, alignment, rigid fixation, bone grafting, and early motion have remained current.

Since our initial series published in 2003, we have treated an additional 10 patients. To date, this remains the largest series in the literature. The combined patient cohort with follow-up at least until complete healing now includes 32 male and 30 females with an average age of 48 years (range 16–88). The average length of time between injury and index surgery was 21 months (range 2–204). Indications for referral were pain, loss of function, instability, or a combination. Thirty-one nonunions were supracondylar, 6 were transcondylar, 4 intercondylar, 13 T-type, 6 medial condylar, and 2 lateral condylar. A total of 47 patients had undergone previous internal fixation with an average number of previous operations of 1.5 (range 1–9). The operative approach used at the index procedure (leading to healing) was an olecranon osteotomy in 36 patients; in 26, a triceps splitting, reflecting, or paratricipital approach was done. Six patients had an infection prior to our index surgery. None had an active infection at the time our index surgery. Nineteen had an isolated preoperative ulnar neuropathy and two had a radial nerve deficit. All patients were followed by the respective surgeons until healing.

All but one patient healed their nonunion. Average time to union was 6.8 months (range 2–45). The average range of motion (ROM) at latest FU was 86° (range 10–140). Complications included two superficial infections (successfully treated with antibiotics), two deep infection (for which irrigation and debridement and antibiotics), and new ulnar neuropathies in five patients. Two patients were found to have positive intraoperative cultures and were treated with 6 weeks antibiotics, which eradicated the infection. One patient developed a compartment syndrome caused by anasarca and recovered completely after fasciotomy. Another patient developed a radial and median neuropathy based on swelling in a radiated elbow with very constrictive soft tissue. He underwent emergent nerve releases. The median nerve recovered completely, but the radial nerve did not recover. Six patients (10%) underwent additional surgery after healing. Four patients underwent hardware removal, one underwent hardware removal and removal of heterotopic bone, and one underwent ulnar neurolysis.

## Discussion

A distal humeral nonunion often leads to a flail and painful arm. There are very few contraindications for surgical treatment. In the English literature, since the early 1980s, 24 publications have been published specifically on the treatment of distal humeral nonunions [1–24]. Most series describe the results after internal fixation [1–3, 8–12, 19, 21, 23], whereas two small subgroups used total elbow replacement [6, 7, 13, 15, 17, 18] or thin wire (Ilizarov) fixation [5, 20]. A summary of the pertinent literature to date is given in Table 1 and illustrates that success rates of formal open reduction and internal fixation of a distal humeral nonunion are high with acceptable complications. It should be noted that all studies were level IV evidence. As this is a rather unusual clinical problem, it is very unlikely that prospective trials will ever be done. Union rate when treated with open reduction and internal or thin wire fixation in these studies ranged between 80 and 100% (average 93%) with a total range of flexion-extension motion arc at latest follow-up reported between 71 and 102° (average 91°).

**Table 1:** Literature review

Author(s)	Year	Patients (*N*)	Procedure or approach	Average age (range)	Follow-up (range)	Success rate, percentage	Results	Level of evidence
Mitsunaga et al.	1982	25	Posterior or lateral, ORIF, graft in 23, ex-fix in 2	43	Avg 2 years. 5 months (1–6 years)	88% united; although 6/22 had multiple operations	Avg ROM arc 71°; pain score avg 1.3 (0–4 scale)	IV
Ackerman and Jupiter	1988	20	ORIF in 17, distal humerus allograft in 2, vascularized graft in 1, immobilization in 1	40 (20–70)	Avg 3.6 years (13–108 months)	94% (1 received custom-made TER)	Avg ROM arc 76° (30–130°); Jupiter score exc 5%/good 30%/fair 35%/poor 30%	IV
Sanders and Sackett	1990	5	Posterior approach, decortication, ORIF, graft	56.2 (22–81)	Avg 40 months (24–65)	100%	Avg ROM arc 86° (38–124°); good 40%/fair 40%/poor 20%	IV
Jupiter and Goodman	1992	6	Posterior approach, debridement, release, ORIF, graft	68 (55–85)	Avg 18 months (12–30)	100%	Avg ROM arc 102° (90–110°); Broberg-Morrey good 83%/fair 17%; HSS score exc 33%/good 50%/fair 17%	IV
McKee et al.	1994	7	Posterior or lateral approach, debridement, ORIF, graft	43.1 (25–62)	Avg 20 months (12–30)	100%	Avg ROM arc 97° (65–115°); Morrey Elbow score Avg 83 (63–97); exc 14%/good 58%/fair 28%	IV
Ring et al.	1999	5 (infected 4, contaminated 1)	Thin wire fixation, debridement, (vascularized) graft	40.2 (15–67)	Avg 3.6 years (2–6)	80% (all these needed second procedure)	Avg ROM arc 94° (90–100°)	IV
Parmasivan et al.	2000	8	Transolecranon, arthrolysis, IM nail antegrade, graft	40.7 (20–62)	Avg 32.1 months (22–41)	88%	Avg ROM arc 94° (10–130°)	IV
Helfet et al.	2003	52 (13 delayed union, 39 nonunion)	Posterior approach, release , debridement, ORIF, graft	47 (16–88)	Avg 33 months (3–198)	98%	Avg ROM arc 94° (10–145°)	IV
Ring et al.	2003	15	Posterior, debridement, ORIF, graft (2 vascularized)	60 (26–75)	Avg 51 months (24–130)	80% (5 needed additional procedures; 3 failed and received total elbow prosthesis)	Avg ROM arc 95° (60-130°); Mayo score exc 13%/good 60%/fair 7%	IV
Ali et al.	2005	16	Posterior approach, debridement, ORIF, graft	47 (19–82)	Mean 39 months (8–69)	100% (1 needed additional graft)	Mean ROM arc 96° (45–130°): MEPS mean 88 (50–100); exc 68.7%/good 12.5%/fair 12.5%/poor 6.3%	IV
Beredjiklian et al.	2005	5	Posterior approach, release, debride, ORIF, vascularized graft	48 (29–70)	Avg 15.2 months (9–24)	80% (1 failure because of articular collapse required prosthesis)	Avg ROM arc 94° (80–110°)	IV
Ring and Jupiter	2006	3 osteochondral nonunions	ORIF	35 (18–47)	Avg 33.6 months (27–46)	100%	Avg ROM arc 30-130°; Mayo score Avg 85 (80–95); DASH Avg 19.5 (4–35); ASES Avg 90 (80–95)	IV
Brinker et al.	2007	6 infected distal humerus nonunions	Debridement, shortening via Ilizarov, graft	49.9 (33–77)	Avg 4.1 years (2–7)	100%	Avg ROM arc 81° (range 70–100°); DASH Avg 77 (50–93); SF-12 Avg 44.8 (33.8–53.4); QALY’s 3.8	IV
Allende and Allende	2009	24 (6 active infection)	Posterior or lateral, release, debridement, ORIF, graft	45 (19–73)	Avg 46 months (18–108)	100%	Avg ROM arc 98° (65–125°); DASH Avg 16 (0–36)	IV
Elbow replacement								
Mitsunaga et al.	1982	7	Total elbow TER	60	Avg 2 years 5 months (1–6 years)	29% revision	Avg ROM arc 103°; pain score Avg 1.8 (0–4 scale)	IV
Figgie et al.	1989	14	Semiconstrained elbow replacement	65 (31–77)	Avg 5 years (2–12)	21% revision	Avg ROM arc 100° (65–120°); HSS score Avg 84 (57–100), exc 42%/good 16%/fair 21%/poor 21%	IV
Morrey and Adams	1995	36	Semiconstrained elbow replacement	67.4 (40–89)	Avg 50.4 months (24–127)	13% revision	Mean ROM arc 111°; Broberg-Morrey exc 69%/good 22%/fair 3%/poor 6%	IV
Ramsay et al.	1999	14	Semiconstrained elbow replacement	66 (52–81)	Avg 77 months (25–128)	14% revision	Avg ROM arc 108° (60–140°); MEPI exc 64%/good 21%/fair 15%	IV
Cil et al.	2008	91 (92 elbows) this is the FU study of Morrey and Adams 1995	Semiconstrained elbow replacement	65 (22–84)	Avg 6.5 years (0.5–20.3 years)	5% deep infections, 5% component fractures, 4% periprosthetic fractures, 13% aseptic loosenings	Mean ROM arc 113°, MEPS exc 38%/good 40%/fair 13%/poor 9%	IV
Pogliacomi et al.	2015	20	Semiconstrained elbow replacement	71.9 (54–84)	Avg 5.5 years (3–12.5)	30% complications; 10% implant revision	Mean ROM arc MEPS Exc 60%/good 30%/poor 10%	IV

ORIF=open reduction and internal fixation, IM=intramedullary, Avg=average, TER=total elbow replacement, ROM=range of motion, HSS=Hospital for Special Surgery score, ASES=American Shoulder and Elbow Surgeons score, DASH=Disabilities of the Arm, Shoulder and Hand score, QALY=quality-adjusted life-year score, MEPS=Mayo Elbow Performance score

Historically, arthrodesis of the elbow was considered an alternative for a flail, non-reconstructable joint. However, obtaining bone fusion is difficult when bone stock is limited. As daily activities are limited with a fused elbow, few patients will choose this as salvage.

Distraction of the elbow joint interposing a layer of soft tissue (usually tensor fascia late autograft) was popularized by the Mayo Clinic. The technique is difficult with inconsistent outcomes. Especially, instability and weakness are a concern. Morrey reported on two patients who had their distal humerus nonunion treated by distraction arthroplasty [14]. Only one had a satisfactory result.

Urbaniak’s series of 10 cadaveric elbow transplants describe 4 patients treated with an elbow allograft for a distal humeral nonunion that were followed for 1–6 years [24]. Indications were disabling elbow joint symptoms in patients who refuse an arthrodesis or are not a candidate for prosthetic replacement because of excessive bone loss or young age. This unique series had significant complications of degenerative joint changes resembling neuropathic joints, nonunion of the allograft-host junction, resorption of the allograft, and disease transmission. Given these concerns, it should be considered a salvage procedure.

Elbow replacement is a good salvage procedure in the low-demand, elderly (>70 years) patient [6, 7, 12, 15, 17, 18]. Its drawbacks are a limited lifespan, component fracture, loosening, and subsequent need for revision. Table 1 shows that the revision rates are high between 10 and 29%. Total reported range of motion arc after total elbow replacement is higher, being an average of 107° (range 100–113°) than for those patients treated with internal fixation (average 91°, range 71–102°). Results of prosthetic elbow replacement as salvage after trauma are not as good or predictable when done as a primary procedure. It should be noted that heavy lifting is to be discouraged with a total elbow replacement and young active patients might not accept these limitations. Most studies to date come from a very experienced group at the Mayo Clinic and might not be easily reproduced by others [6, 15, 18]. Indications for replacement rather than internal fixation are (1) a distal humerus that is either too osteopenic or comminuted for reduction and fixation; and (2) extensive cartilage loss as seen in rheumatoid arthritis, posttraumatic arthritis, or ankylosis. The “ideal” candidate is the sensible older patient with good soft tissues and a retained osteopenic fragment with retention of muscular attachments and epicondyles as required for soft tissue balancing.

**Fig. 8:**
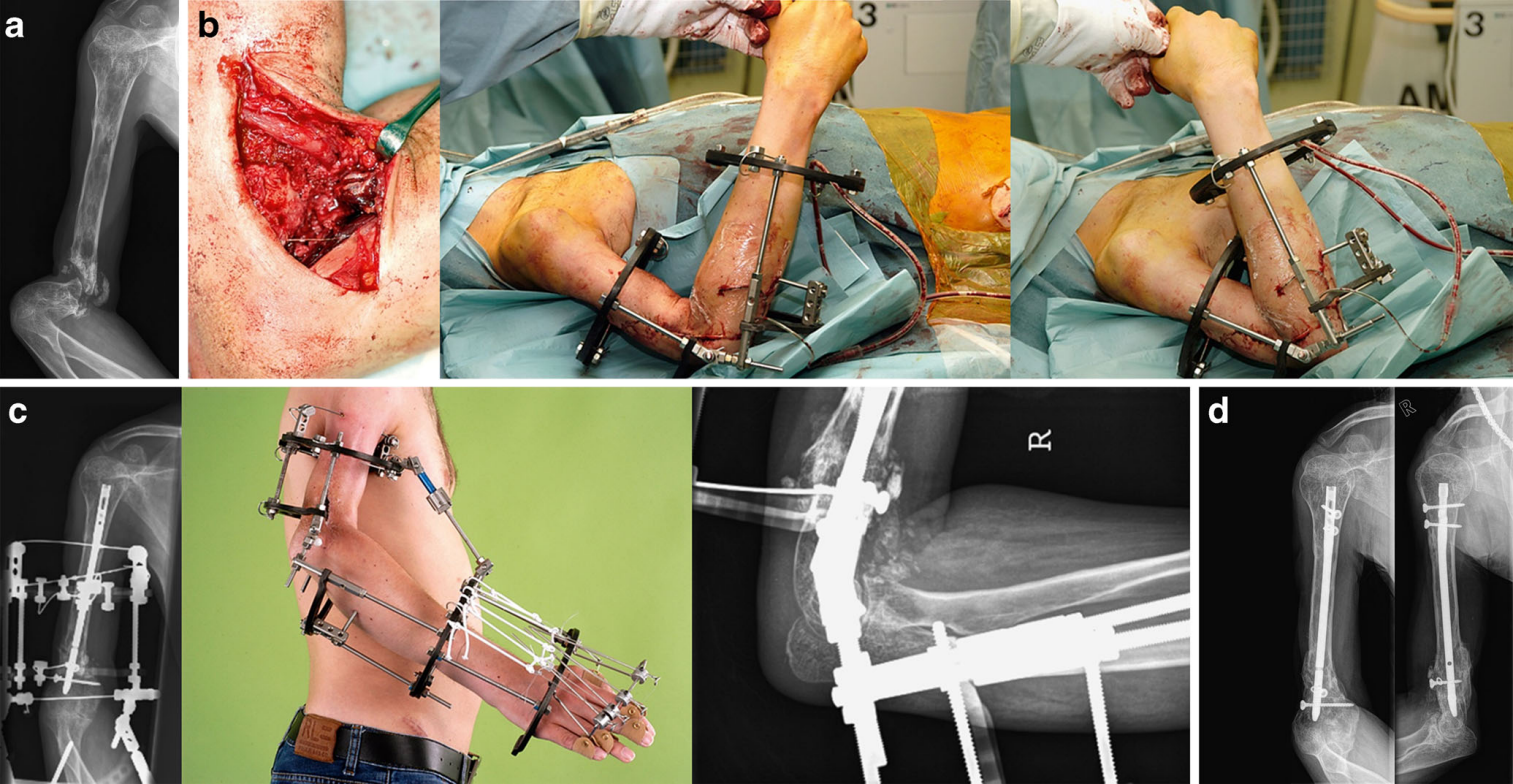
The Ilizarov can be an extremely useful tool in complex cases not amenable to open reduction and internal fixation. A 26-year-old medical student presented with a distal humerus nonunion. As an 8-year-old, he underwent chemotherapy and radiation for an Ewing sarcoma of the humerus. At age 12, he sustained a distal humerus fracture treated with a cast for 2 years. Numerous surgeons were consulted during these years but surgical therapy was felt too risky as his upper arm had essentially remained the same size as when he was 8 years old with a thickened stiff skin and soft tissue cuff around the nonunion. He functioned reasonably well and entered medical school anticipating a career in plastic surgery. During his medical school, he developed increasing pain and instability of the arm and presented to us. Motion was limited to the nonunion site with a stiff elbow joint as seen on the lateral radiograph (**a**). Formal ORIF using an open approach was not an option. We referred the patient to an expert in Ilizarov techniques (Dr. Dror Paley) who agreed to operate in a combined procedure with the authors. Via a minimal approach the nonunion was debrided and an intramedullary nail was placed as an internal strut and an Ilizarov frame with an elbow hinge was then placed (**b**). Autologous bone graft was added locally. AP radiograph, clinical photo and lateral flexion radiograph (*from left to right*) illustrates final construct (**c**). In the next 24 hours, he developed increasing swelling and a median and radial nerve deficit (likely because of anasarca because of compromised lymph outflow). Exploration of the median and radial nerves was done on post-operative day two; additional bone graft was added at 6 months. At that time, the Ilizarov frame was removed and the nail was locked proximally. His nonunion healed as seen in AP and lateral radiographs (*from left to right*), (**d**). The median nerve fully returned; the radial nerve deficit remained complete. Eleven years later, he is pleased with the outcome—despite the radial nerve deficit. There is no pain and his elbow is stable. He is now working as a radiologist and has returned to all athletic activities including downhill skiing.

For infected distal humerus nonunions or those with compromised soft tissues, the Ilizarov technique of thin wire fixation is a good—but difficult—alternative [5, 20]. There is a steep learning curve and patients need to be motivated as the frame is cumbersome. We have limited experience with this technique (Fig. 8). Two small series using Ilizarov fixation for a distal humeral nonunion in the English literature have been published. The series of Jupiter et al. showed an 80% union rate in 5 patients but these patients all needed more than 1 procedure [20]. Brinker’s series presented a single-stage procedure with debridement, release, shortening, Ilizarov frame fixation, and bone grafting. Using this protocol, they reported 100% healing in 6 patients [5]. It should be noted that these were very complex cases where formal internal fixation was not feasible because of soft tissue issues or active infection.

Presumed aseptic nonunions form a subgroup that might be underestimated. It is important always to consider infection as an underlying reason for nonunion, even if there are no obvious clinical signs of infection. Especially, *Propionibacterium acnes* is known to be associated with upper extremity nonunions. Always obtain deep cultures and customize treatment in collaboration with an infectious disease specialist.

In conclusion, internal fixation and bone grafting remains the treatment of choice for a nonunion of the distal humerus. Our results and those of others have shown careful preoperative planning, extensile approach, thorough debridement, and release of scarred soft tissues are essential to a successful reduction and optimal realignment. Rigid fixation with versatile locking plates and liberal use of bone graft will maximize the union rates and the reestablishment of a functional elbow in most patients.

## Electronic supplementary material

ESM 1 (PDF 1224 kb)

ESM 2 (PDF 1224 kb)

ESM 3 (PDF 1224 kb)

ESM 4 (PDF 1224 kb)
